# Biochemical and antioxidant activity of wild edible fruits of the eastern Himalaya, India

**DOI:** 10.3389/fnut.2023.1039965

**Published:** 2023-03-01

**Authors:** Heiplanmi Rymbai, Veerendra Kumar Verma, Hammylliende Talang, S. Ruth Assumi, M. Bilashini Devi, Vanlalruati, Rumki Heloise CH. Sangma, Kamni Paia Biam, L. Joymati Chanu, Badapmain Makdoh, A. Ratankumar Singh, Joiedevivreson Mawleiñ, Samarendra Hazarika, Vinay Kumar Mishra

**Affiliations:** ICAR Research Complex for NEH Region, Umiam, India

**Keywords:** wild edible fruits, bioactive compounds, pigmentation, processing, value addition, livelihood, diversity, conservation

## Abstract

The eastern Himalayas, one of the important hotspots of global biodiversity, have a rich diversity of wild edible fruit trees. The fruits of these tree species have been consumed by the tribal people since time immemorial. However, there is limited information available on the biochemical and antioxidant properties of the fruits. Therefore, the present investigation was undertaken to study the physico-chemical and antioxidant properties of the nine most important wild fruit trees. Among the species, *Pyrus pashia* had the maximum fruit weight (37.83 g), while the highest juice (43.72%) and pulp content (84.67%) were noted in *Haematocarpus validus* and *Myrica esculenta*, respectively. Maximum total soluble solids (18.27%), total sugar (11.27%), moisture content (88.39%), ascorbic acid content (63.82 mg/100 g), total carotenoids (18.47 mg/100 g), and total monomeric anthocyanin (354.04 mg/100 g) were recorded in *H. validus*. *Docynia indica* had the highest total phenolic content (19.37 mg GAE/g), while *H. validus* recorded the highest total flavonoids and flavanol content. The antioxidant activities of the different fruits ranged from 0.17 to 0.67 IC_50_ for DPPH activity and 3.59–13.82 mg AAE/g for FRAP. These fruits had attractive pigmentation of both pulp and juice and were a good potential source for the extraction of natural edible color in the food industry. The fruits also possess high market prices; *Prunus nepalensis* fetched $ 34.10–$ 141.5 per tree. Therefore, these fruits are rich sources of antioxidants, pigments and have a high market value for livelihood and nutritional security.

## 1. Introduction

The eastern Himalayan region of India has a diverse agro-climate, ranging from tropical to alpine, and receives very high rainfall. The region is an important part of the Indo-Myanmar biodiversity hotspot of the world (1). The diverse agro-climatic conditions of this region offer immense scope for the evolution and development of different wild edible species. Of the 800 wild edible tree species found in India, about 300 are consumed by the hill populace of this region alone. Therefore, the region is considered a reservoir of several crop species, including wild relatives, grown naturally in the forests and also in the backyards of the local tribes. The economy of this region is basically rural based; agriculture and allied sectors play a predominant role. The fruits collected from the forest as well as from their own land are consumed locally and also sold in local markets at a premium price. Recently, several trainings and demonstrations have been conducted by various government agencies to impart knowledge and skills to the local people, resulting in improved resource utilization and entrepreneurial skills. Several value-added products, such as wine, vinegar, jam, jelly squash, RTS, pickles, etc., of these wild fruits are prepared and marketed by self-help groups (SHGs) and entrepreneurs. However, the availability of value-added products on the market is still lacking due to poor commercial production.

These fruit plants are found in tropical, subtropical, and temperate regions of the Indian subcontinent, East Asian nations, South East Asian nations, and European nations, which suggests that they are adaptable to a wider range of environments (Table 1). These fruits have been important constituents of diet and health care and have contributed significantly to the livelihood security of the local people over centuries (2). Locally, these crops are also used to extract natural pigments (3); and ethnobotanical uses of fruits in the treatment of cancer (4); fever, cough, and jaundice, gastrointestinal, respiratory, and cardiovascular (5); anti-obesity (6); cognitive boosting properties, liver health, and reducing fatty liver buildup (7); leaves of *Pyrus pashia* were used as fodder (8) and the Monpa community of Tawang, Arunachal Pradesh, India, used extracts of *P. pashia* as butter tea beverages (9). This could be due to the nutraceutical properties of these fruits. It has been proven that ingestion of natural antioxidants from fruit sources, such as polyphenols, have significant anticarcinogenic, antipyretic, anticoagulant, anti-inflammatory, and hypoglycemic properties (10). This might be attributed to the powerful antioxidant capacity of polyphenols and their additives, and their synergistic effects with associated bioactive constituents. Such constituents provide protection to the cellular system against oxidative impairment, which consequently reduces the oxidative stress in the human body (11). In addition, fruit-based natural antioxidants are also fascinating because of their safety and wide applications in the cosmetic, pharmaceutical, and food industries as alternative sources to synthetic antioxidants (12). In spite of these potential applications, research on these crops is at an infant stage, although morphological characterization of some fruit crops, such as *Prunus nepalensis*, *Elaeagnus latifolia*, *Pyrus pashia*, and other wild edible fruits has been described (13–15). However, their nutraceutical properties have not yet been scientifically assessed. Hence, the present study was carried out to determine the biochemical and antioxidant properties of popular wild edible fruit trees grown in the eastern Himalayas of India. The information generated could lead to a better understanding of the potential functional food sources and an increased consumption of these fruits. This, in turn, could have a significant impact on the most vulnerable tribal population’s long-term economic, nutritional, and health system in the near future.

**TABLE 1:** Habitat, distribution, and utilization of wild edible fruit crops of the eastern Himalayas, India.

Crops	Habitat and distribution	Uses
*Prunus nepalensis* Ser. (Family: Rosaceae and Vernacular name: Sohiong)	● Habitat: Subtropical and temperate Himalayan regions at an altitude of 800–3,000 m amsl. ● Native: Eastern Himalayas including the Khasi and Jaintia Hills of Meghalaya, India. ● Domestication status: Growing wild and semi cultivated. ● Distribution: It is found in Meghalaya, and other parts of Himalayas India (2). The species also found in Bhutan, Nepal, Myanmar, and China (16).	● Edible portion: Epicarp and mesocarp. ● Dessert purpose: Fresh fruits. ● Processing: The processed products prepared from this fruit are ready to serve drink (RTS), squash, candy, powder, wine, tooty fruity (Sohiong + chow chow). The products developed retained the natural purple color of the fruits for longer period (up to 1 year). ● Others: Fruits are used as astringent, leaf as diuretic agent against edema (17).
*Elaeagnus latifolia* L. (Family: Elaeagnaceae and Vernacular name: Sohshang)	● Habitat: Thrives well in open forest and swamps of the foothills track of Eastern Himalayas up to elevations of 2,600 m amsl. ● Native: The lower hill tracks of Himalayas considering its wider genetic variability. ● Domestication: The shrub mostly found in back yard as semi-wild and semi cultivated (13). The genus is also reported to be cultivated in warmer parts of southern Europe, North America and Vietnam (18). ● Distribution: Subtropical and temperate Himalaya including Myanmar and China.	● Edible portion: Epicarp and mesocarp of fleshy drupe fruits. ● Dessert purpose: Fresh fruits. ● Processing: pulp used for preparation of pickle, jam, jelly and leather. Refreshing drink prepared from fruit juice possess attractive reddish or pinkish color. ● Others: Fruits are astringent (19) and found to reverse the growth of cancers (4). Pulp also used for dye extraction and seed as source of oil. The plant also possesses ornamental values.
*Elaeagnus pyriformis* Hook. f. (Family: Elaeagnaceae and Vernacular name: Sohkhlur)	● Habitat: Thrives well in open forest up to elevations of 2,600 m amsl. ● Native: Foot hill tracks of the Eastern Himalayas India. ● Domestication: The shrub mostly found in forest areas. ● Distribution: Subtropical and temperate Himalaya including Myanmar and China.	● Edible portion: Epicarp and mesocarp of fleshy drupe fruits. ● Dessert purpose: Fresh fruits. ● Processing: Processed products such as pickle, jam and jelly were prepared from pulp. Fruit beverage develop an attractive attractive reddish or pinkish color. ● Others: Fruits are capable of reducing cancer and reversing the growth of cancers (4).
*Myrica esculenta* Buch. -Ham. ex D. Don. (Family: Myricaceae and Vernacular name: Sohphie bah)	● Habitat: It flourish well in mixed forests of *Pinus* sp., *Quercus leucotrichophora* and marginal lands of nitrogen depleted soils up to an altitude of 2,000 m amsl in the sub-tropical Himalayas (20). ● Native: North east and northern India, southern Bhutan and Nepal. ● Domestication: Semi-cultivated. ● Distribution: In the Indian subcontinents, it is confined in the sub-tropical Himalayas ranging from Punjab eastward to Assam. It was also found in the temperate and sub-tropical regions of China of both hemispheres except Australia (21).	● Edible portion: Epicarp and mesocarp of drupaceous fruits. ● Dessert purpose: Fruits are edible as fresh at all stages of its growth. ● Processing: Fruits are used for making refreshing drink and pickle. The extracted juice emits a very attractive sparkling red color. ● Others: Fruit juice are used for treatments of jaundice in Khasi Hills (22), fever in Khasi Hills, Vietnam, South China (23), Ulcer, and Anthelmintic in Himachal (24, 25), Bronchitis, dysentery in Nepal (26). The bark is used as aromatic, tonic for rheumatism, astringent, carminative, asthma, odontalgia, diarrhea, lung infection, fever, cough, bronchitis, dysentery, antiseptic indigenous medicine (27, 28). Tannin extract from the barks are used as a yellow dyeing agent (29).
*Myrica nagi* Thunb. (Family: Elaeagnaceae and Vernacular name: Sohphienam)	● Habitat: The tree is evergreen in the sub-temperate of mid-hill and hill tracks of the Himalayas up to 2,100 m amsl. ● Native: Eastern Himalayas, India. ● Domestication: Found wild in the forest. ● Distribution: It is found in the mid-Himalayas of India including the Khasi Hills. It is also found in Bangladesh, Singapore, Malayan islands, China and Japan (30).	● Edible portion: Epicarp and mesocarp of drupaceous fruits. ● Dessert purpose: Fruits are edible as fresh at all stages of its growth. ● Processing: Pulp are used for preparation of refreshing drink and pickle. The juice possesses a very attractive sparkling pink color. ● Others: Bark powdered is used against dysentery (31).
*Baccaurea sapida* (Roxb.) Müll.Arg. (Family: Phyllanthaceae and Vernacular name: Sohramdieng/ Sohmyndong)	● Habitat: Grow favorably in moist tropical up to an altitude of 900 m asml. ● Native: South East Asian region. ● Domestication: Growing wild and semi-cultivated in the sub-Himalayan tract of eastern India. It is cultivated in China, Myanmar, Thailand, Vietnam, and Malaysia (18). ● Distribution: It is found from Bihar to Arunachal Pradesh and in the lower hills and valleys (of Meghalaya, Assam, Nagaland, Manipur, Mizoram, and Tripura), Orissa and Andaman and Nicobar Islands. Globally, its distribution is from Indo-Malaysia to the West Pacific.	● Edible portion: Arils is very delicious at ripening stage. ● Dessert purpose: Fresh fruits at ripened stage. ● Processing: Products prepared are squash, RTS, wine, jam and jelly due to its rich sources of pectin (14.1%). Fruit rinds are also used for making pickle. ● Others: Fruits and leaf produced dye of chocolate color which can be used as natural colorants in processed products. Seed was used to extract annatto dye (4.8–6.0%) for coloring of silk, cotton and other textile materials for orange-red color (3). Fruit juice are used for treatment against arthritis, abscesses, injuries, and constipation (32).
*Pyrus pashia* Buch.-Ham. ex D. Don. (Family: Rosaceae and Vernacular name: Sohjhur)	● Habitat: It thrives well in moist soil up to an elevation 2,700 m amsl. It is tolerant to drought and atmospheric pollutants. ● Native: Southern Asia. ● Domestication: It is cultivated in Khasi and Jaintia Hills as back yard and border tree crops. ● Distribution: It is distributed from East Afghanistan, North Pakistan through Himalaya to Vietnam.	● Edible portion: Thalamus or receptacle of pome fruits. ● Dessert purpose: Fruit are eaten fresh, and preferred for its sweetness and grittiness. ● Processing: ● Others: Fruit juice is astringent and diuretic (33) used against constipation (34), dysentery (35), leishmaniasis (36), eye problems (37), digestive disorder, sore throat, irritability, abdominal pain, anemia, curing eye disorder (38) and curing of gastrointestinal, respiratory and cardiovascular related problems (5). Decoctions of dried fruits with other plant parts improves in spleen and stomach functions (39). Leaf extract as a tonic for hair loss, treatment of digestion related ailments and possess antimicrobial activity (40). Staining of crushed leaves in palm, feet and nails improve cosmetic appearance (41). The barks are used to treat digestive disorders (42), sore throat, fever, peptic ulcer, gastric ulcer and typhoid fever (43). Leaves are used as fodder for goats and sheep (8) and it extracts as butter tea beverages by the Monpa community of Tawang, Arunachal Pradesh, India (9). The fruit is added to cattle fodder to enhance milk production (44). Seedlings can be used as rootstock for pear and quince grafts (28).
*Docynia indica* (Wall.) Decne. (Family: Rosaceae and Vernacular name: Sohphoh/shoptet)	● Habitat: Grows well in moist soil, open places, and upland temperate to subtropical at an elevations of 700–3,000 m amsl. ● Native: Eastern Himalayas of India through Nepal to South Central China. ● Domestication status: Growing in the back yard and also naturally in forest areas of Khasi and Jaintia Hills of Meghalaya, India. In Northern Vietnam, the species is domesticated in large area (3,000 ha) with a production of 6,500 tons of fruit (45). ● Distribution: Southern foothill tracks of the Himalayan range from Pakistan through India, Nepal, Bhutan, and Bangladesh to the mountains of northern Myanmar, Thailand, Laos, Vietnam, and southern China.	● Edible portion: Mesocarp of matured fruits. ● Dessert purpose: The fully ripe fruit is eating as fresh, while, the half ripe fruits are consumed as fresh with salt. ● Processing: The fruits soaked in brined solution or boiled with syrup and sundried. Pulp is used as raw materials for production of juice, tea, vinegar, wine, pickles and jelly (13, 45). ● Others: Fruits are used as natural remedy in treatment of infectious diseases, obesity (6), digestive problems and possess hypoglycemic and hypolipidemic properties (46). Fruit extract possesses antioxidant and antibacterial properties which have industrial potential as food preservative (47). The species as rootstock imparting semi-dwarf in apple.
*Haematocarpus validus* (Miers) Bakh. f. ex Forman (Family: Menispermaceae and Vernacular name: Sohsnam)	● Habitat: An evergreen vine and prefers the tropical to subtropical conditions with a large number of herbaceous undergrowth on a hilly landscape up to altitude 1,250 m amsl. ● Native: Eastern Himalayas India to West Java. ● Domestication status: Growing in the forest naturally. ● Distribution: The geographical distribution of this species is limited and it has been categorized as critically endangered in Meghalaya (48). In India, it is found in Meghalaya, Tripura, Assam, Arunachal Pradesh, Sikkim, West Bengal, and Andaman and Nicobar Islands. It is also found in Bangladesh, Indonesia, Pakistan, and South east Asian.	● Edible portion: Mesocarp of ripened fruits. ● Dessert purpose: Ripened fruits. ● Processing: Immature fruits are used for value-added products such as squash, pickles, and chutney. ● Others: Fruits and seeds were consumed for treatments against anemia, roots for curing itching and tender shoots for treatment of jaundice (49), while leaf decoction was used against body ache (50). The fruit is a rich source of choline for cognitive boosting properties, liver health and reduce fatty liver build up (51). Fruits are rich source of purple to bright reddish pigmentation used for dyeing local handicrafts and may have potential application as natural colorant in food industries.

## 2. Materials and methods

### 2.1. Materials and experimental site

**FIGURE 1:**
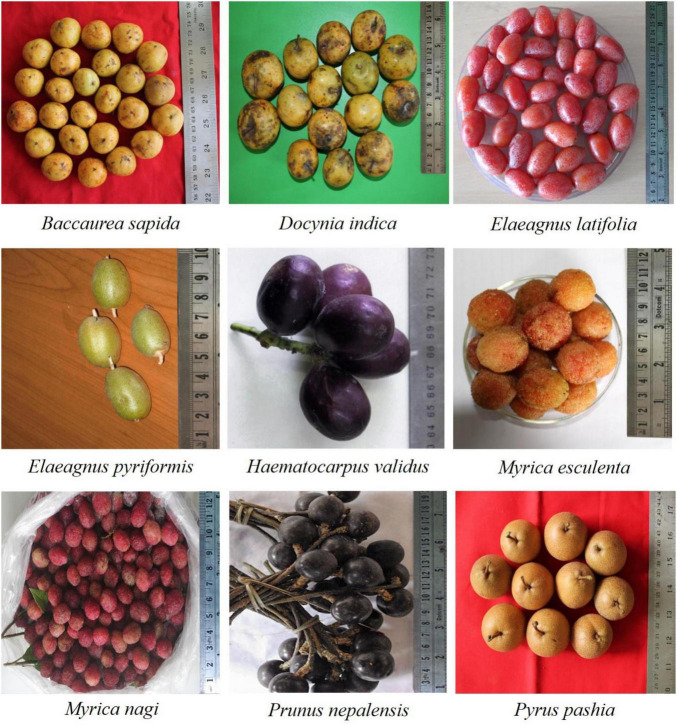
Fruits of wild edible plants grown in the eastern Himalayas, India.

**FIGURE 2:**
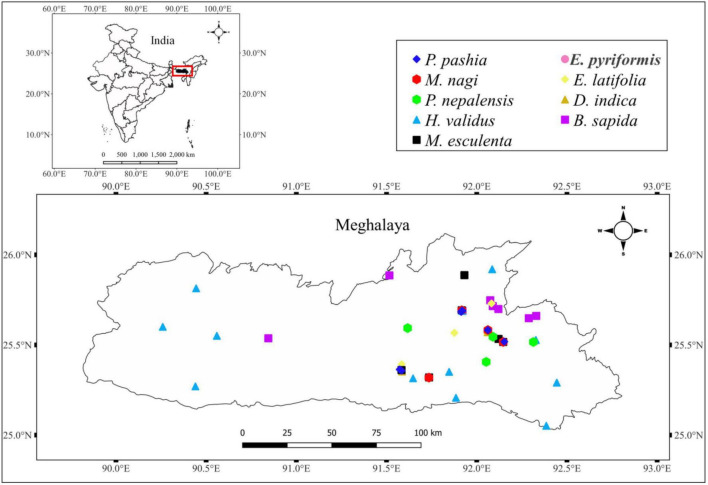
Collection sites of wild edible fruits grown in the eastern Himalayas, India (Generated by subjected the global positioning system (GPS) data to quantum geographic information system (QGIS) version 3.20.1).

The fruits of wild edible plant species such as *Baccaurea sapida* (Roxb.) Müll. Arg., *Docynia indica* (Wall.) Decne., *Elaeagnus latifolia* L., *E. pyriformis* Hook. f., *Haematocarpus validus* (Miers) Bakh. f. ex Forman., *Myrica esculenta* Buch. -Ham. ex D. Don*., Myrica nagi* Thunb., *Prunus nepalensis* Ser., and *Pyrus pashia* Buch.-Ham. ex D. Don. grown in the forests and/or backyards, were collected for the study (Figure 1). The collection was made from various locations in the region, particularly the Khasi Hills, Jaintia Hills, Ri Bhoi, and Garo Hills, distributed between 20.1–26.5°N latitude and 85.49–92.52°E longitude with altitude ranging from 100 to 2,000 m amsl (Figure 2). The collected fruits were analyzed for different biochemical and functional attributes at the ICAR Research Complex for the North Eastern Hill Region, Umiam, Meghalaya, India, during 2019–2020.

### 2.2. Quantitative analysis

Twenty-five ripe fruits of each species were used for carrying out all the physical and biochemical analyses. Fruit samples were harvested at an appropriate maturity. The harvested fruits were washed with distilled water, wiped with tissue paper, and kept at room temperature for 10 min to remove the adhering water before analysis. The parameters, *viz*., fruit, and seed weights, were determined using an electronic balance (Adair Dutt-1620C). Fruit length and diameter were measured using a digital caliper (Code 1108-150). The pulp recovery percentage was estimated using the following formula:


$$\mathrm{Pulp}\mathrm{Recovery}\left(\%\right)=\frac{\mathrm{Pulp}\,\mathrm{weight}\,\left(g\right)}{\mathrm{Fruit}\,\mathrm{weight}\,\left(g\right)}\times 100$$

### 2.3. Determination of biochemical attributes

Biochemical parameters such as total soluble solids (TSS) were determined using a hand-held refractometer (HI 96801) and titratable acidity, ascorbic acid, reducing sugars, and total sugars were analyzed according to Rangana (52). The moisture content of the fruits was determined gravimetrically as per the method of Akter et al. (53) and Raaf et al. (54). The fresh fruit samples were weight before and after drying in a hot air oven (thermostatically controlled, Model–IC7). About 20 g finely shredded fresh sample was placed in a clean and dried crucible with a cover, and accurately weighed on an electronic weighing balance (Model–AUX220). The samples were dried in the oven at 105°C for 24 h or until a constant weight was achieved for two consecutive weights. Following drying, the crucible was cooled in a desiccator. The moisture content (MC) was calculated as below and expressed as a percentage.


$$\text{MC}\left(\%\right)=\frac{\mathrm{W1}-\mathrm{W2}}{\mathrm{W1}}\times 100$$

Where W_1_–fresh weight

W_2_–dried weight

### 2.4. Measurement of total carotenoids

Total carotenoids were determined as per the method of Chen et al. (55). The extraction of carotenoids was carried out according to the method developed by Chen et al. (55). Pulp (10 g) was placed in a vessel, protected from light, and mixed with 50 mL of extraction solvent (hexane/acetone/ethanol: 70:15:15, v/v/v). The mixture was stirred for 1 h using an orbital shaker. About 5 mL of a 40% KOH in methanolic solution were added, and the solution was saponified at 25°C in the dark for 2 h. Subsequently, 30 mL of hexane were added, the mixture was shaken vigorously, and the upper layer was collected. The lower layer was extracted twice, and the supernatant was also collected and filtered through sodium sulfate powder to remove traces of water. The supernatant obtained was pooled and stored at −80°C under a nitrogen atmosphere (99.9% purity) in the dark until analysis. The total carotenoid content of the extracts was measured using a UV-Visible spectrophotometer at 450 nm. A calibration curve (0–50 ppm) was prepared using β-carotene as the standard and the results were expressed as mg β-carotene/100 g sample.

### 2.5. Determination of functional attributes and antioxidant activity

#### 2.5.1. Preparation of fruit extract

The pulp (5 g) of each fruit was grinded, and 50 mL of aqueous methanol was added at ambient temperature. The mixture was incubated for 1 h at room temperature with continuous magnetic stirring at 200 rpm and centrifuged at 1,000 *g* for 20 min. The supernatant was collected and stored at −20°C until analysis. The aliquot was used for assessments of total phenolic content, total monomeric anthocyanins, total flavonoids, total flavonol, DPPH free radical scavenging capacity, and FRAP reducing power.

#### 2.5.2. Determination of total phenolic content

The crude extracts were estimated for total phenolic content using the Folin–Ciocalteu procedure as per the method of Singleton and Rossi (56). About 1 mL of the extract was transferred to 2 mL of Folin–Ciocalteu reagent (1:10 v/v distilled water). After 10 min, 1.6 mL (7.5%) of sodium carbonate was added. The mixture was vortexed for 15 s before being left to stand for 30 min at room temperature to develop its color. The absorption was measured at 743 nm in a UV-visible spectrophotometer (Model: UV 3200). The concentration of polyphenols in samples was derived from a standard curve of Gallic acid, and the total phenolic content was expressed as Gallic acid equivalents (GAE) in mg/g of pulp.


TPC⁢(mg⁢GAE/g⁢fw)

$$\;=\frac{\mathrm{Conc}.\mathrm{of}\,\mathrm{GA}\,\mathrm{from}\,\mathrm{Std}\,\mathrm{curve}\times \mathrm{vol}.\mathrm{of}\,\mathrm{ext}\,\text{ract}\times 100}{\mathrm{Weight}\,\mathrm{of}\,\mathrm{the}\,\mathrm{sample}}$$

#### 2.5.3. Determination of total monomeric anthocyanin content

Total monomeric anthocyanin was determined as per the procedure of Giusti and Wrolstad (57); Lako et al. (58). About 0.4 mL of the extract solution was taken, and 3.6 mL of the corresponding buffer; pH 1.0 buffer (potassium chloride 0.025 M) and pH 4.5 buffer (sodium acetate, 0.4 M) was added. The absorbance of each solution was taken against a blank in a cuvette with a 1 cm path length at 510 nm and 700 nm using a UV-Visible spectrophotometer. Total monomeric anthocyanin pigment concentration was expressed as cyanidin-3-glucoside equivalents (mg cyd-3-gluE/100 g) as follows:


Anthocyanin⁢Pigment⁢(mg⁢cyd⁢-⁢3⁢-⁢gluE/100⁢g⁢fw)

$$\;\;=\frac{\text{A}\times \text{MW}\times \text{DF}\times 1000}{\varepsilon \times \text{l}}$$

Where A = (A_510nm_ – A_700nm_) pH 1.0 – (A_510nm_ – A_700nm_) pH 4.5;

MW (molecular weight) = 449.2 g/mol for cyanidin-3-glucoside (cyd-3-glu);

DF = dilution factor established in D;

*l* = pathlength in cm;

ε = 26,900 molar extinction coefficients for cyd-3-glu; and 1,000 = factor for conversion from g to mg.

#### 2.5.4. Measurement of total flavonoids

The total flavonoid content of extracts was estimated using Aluminum chloride (AlCl_3_) colourimetric assay as previously described by Zhishen et al. (56). About 0.3 mL of 5% NaNO_2_ was added to 1 mL extract. After 5 min, 0.3 mL of 10% AlCl_3_.6H_2_O was added, and incubated for 5 min. About 2 mL NaOH (1M) was added, and the final volume of the solution was adjusted to 5 mL with distilled water. After 15 min of incubation, the mixture turned to pink and the absorbance was measured at 510 nm (UV-visible spectrophotometer, Model: UV 3200). Total flavonoid content was presented as mg quercetin equivalent per gram (mg QE/g).


Flavonoid⁢content⁢(mg⁢QE/g⁢fw)

$$\;=\frac{\mathrm{Conc}.\mathrm{of}\,Q\,\mathrm{from}\,\mathrm{std}\,\mathrm{curve}\times \mathrm{volume}\,\mathrm{of}\,\mathrm{extract}}{\mathrm{Wegiht}\,\mathrm{of}\,\mathrm{the}\,\mathrm{sample}}$$

#### 2.5.5. Determination of total flavonols

Total flavonols in the fruit sample extracts were determined according to the method of Miliauskas et al. (59). 2 mL of 2% AlCl_3_ and 6 mL (5.0%) sodium acetate solutions were added to 2.0 mL of extract. The mixture was incubated at 25°C for 2.5 h and absorption at 440 nm (UV-visible spectrophotometer, Model-UV 3200) was read. Total flavonol content was expressed as quercetin equivalent (mg QE/g).


Flavonol⁢content⁢(mg⁢QE/g⁢fw)

$$\;=\frac{\mathrm{Conc}.\mathrm{of}\,Q\,\mathrm{from}\,\mathrm{std}\,\mathrm{curve}\times \mathrm{volume}\,\mathrm{of}\,\mathrm{extract}}{\mathrm{Weight}\,\mathrm{of}\,\mathrm{the}\,\mathrm{sample}}$$

#### 2.5.6. Measurement of DPPH free radical scavenging activity

The free radical scavenging activity of the fruit extracts was estimated with the DPPH (1, 1-diphenyl-2- picrylhydrazyl) method (60). Ascorbic acid was used as a reference standard. 100 μL of aliquot was transferred to test tubes, to which 3.9 mL of freshly prepared DPPH solution (25 mg/L in methanol) were added. The mixtures were then thoroughly mixed and allowed to stand for 30 min. The absorbance was measured at 517 nm (UV-visible spectrophotometer, Model: UV 3200). The percent scavenging activity of DPPH was calculated using the following formula:


$$\mathrm{DPPH}\mathrm{Scavenging}\mathrm{activity}\left(\%\right)=\frac{\mathrm{Ac}-\mathrm{At}}{\text{Ac}}\times 100$$

Where, Ac is the absorbance of the control reaction and At is the absorbance of the sample of the extracts. The antioxidant activity of the extract was expressed as IC_50_ (the concentration of fruit sample required to decrease the absorption at 517 nm by 50%). The IC_50_ value was expressed as the concentration in milligram of extract per mL that inhibited the formation of DPPH radicals by 50%.

#### 2.5.7. Measurement of FRAP reducing power

The reducing power of the extracts was assessed as per the method of Oyaizu (61). About 100 μL of fruit extracts were mixed with phosphate buffer (2.5 mL, 0.2 M, pH 6.6) and 1% potassium ferricyanide (2.5 mL). This mixture was incubated at 50°C for 20 min, to which 2.5 mL aliquots of trichloroacetic acid (10%) was added. The content was centrifuged at 3,000 rpm for 10 min. The upper layer of the solution (2.5 mL) was extracted and mixed with 2.5 mL of distilled water and 0.5 mL of freshly prepared ferric chloride solution (0.1%). Then the measurement of absorbance was recorded at 700 nm (UV-visible spectrophotometer, Model: UV 3200) and the reducing power was expressed in terms of ascorbic acid equivalent (AAE) in milligram per gram of extract (mg AAE/g).

### 2.6. Color, season of availability and market price of fruits

Color measurements of ripened fruits of different fruit tree species were carried out using a Color Hunter meter (HunterLab Color Quest XE). The instrument was calibrated using the black and white tiles. The value was expressed as L* values indicated lightness (black, L* = 0 and white, L* = 100), a* values indicated redness-greenness (red, a* = 100 and green, a* = −100), b* values indicated yellowness-blueness (yellow, b* = 100 and blue, b* = −100). The observation was replicated thrice for each sample. Observations were taken at the base, middle, and apex of fruits at an equidistant space under the aperture of the color meter. Through image analysis, an Android application (Color Grab version 3.9.2) was used to determine the color of fruit juice. A local market survey in Shillong city and 10 weekly markets in Khasi and Jaintia Hills were conducted. Informants (60 no.) were randomly selected among the local vendors and farmers for data collection on period of fruit availability in the markets and the market price of fruits. The selection of key informants was done with the help of village workers and elders as per the ethnoecological methods of Martin (62). The yield of fruits per tree was determined by counting the number of fruits per tree at harvest and multiplying it by its fruit weight, expressed in kg per tree.

### 2.7. Statistical analysis

The replicated (three of each parameter) data were analyzed using statistical package for the social sciences (SPSS) (Version 14.0) software, and the data were presented as mean ± SE using one-way ANOVA (*p* < 0.05) of Tukey’s HSD (honestly significant difference) test. The possible relationship between antioxidant compounds and antioxidant activity was analyzed through Pearson’s correlation coefficient. Using quantum geographic information system (QGIS) version 3.20.1, a map of the collection sites was created subjecting the global positioning system (GPS) data.

## 3. Results and discussion

### 3.1. Physico-chemical characteristics

The biochemical traits of fruits contribute to the consumer’s perception of quality traits, including those associated with taste, mouth feel, and appearance. The results revealed a significant variation among the fruit morphological and biochemical characteristics of different wild edible fruit species (*p* < 0.05, Table 2). The maximum fruit length ranged from 4.38 cm in *H. validus* to 1.52 cm in *M. nagi*; fruit diameter (1.23 cm in *M. nagi* to 4.39 cm in *P. pashia*); fruit circumference (12.38 cm in *P. pashia* to 3.64 cm in *M. nagi*); fruit weight (7.32 g in *E. pyriformis* to 37.83 g in *P. pashia*); fruit volume (39.89 cm^3^ in *P. pashia* to 7.32 cm^3^ in *E. pyriformis*); juice content (21.22% in *M. nagi* to 43.72% in *H. validus*) and pulp content (56.69% in *B. sapida* to 84.67% in *M. esculenta*). The significant differences in fruit physical characteristics indicated greater variability among fruit crops. The maximum fruit weight was observed in *P. pashia*, followed by *D. indica, H. validus*, and *E. latifolia*; juice content was recorded in *H. validus*, followed by *M. esculenta, B. sapida*, and *E. pyriformis*; and pulp content (>70%) in *M. esculenta*, followed by *M. nagi, P. pashia, P. nepalensis, D. indica, and H. validus*. Similarly, there was a significant difference (*p* < 0.05) among fruits for biochemical attributes as given in Table 3. The moisture content was the highest in *H. validus* (88.39 ± 1.85%) and the lowest in *P. pashia* (73.75 ± 1.88%). The determination of moisture content in food is considered to be one of the most important assays since moisture greatly influences the physical properties and stability of the food (63). The total soluble solids (TSS) was the maximum in *H. validus* (18.27 ± 1.49%) and the minimum in *M. esculenta* (5.83 ± 0.30%). The titratable acidity was the highest in *M. esculenta* (3.32 ± 0.06%), followed by *E. latifolia* (2.68 ± 0.04%) and the lowest in *P. pashia* (0.31 ± 0.03%). Total sugar ranged from 3.26 ± 0.05% in *E. latifolia* to 11.27 ± 1.26% in *H. validus*. Reducing sugar content ranged from 1.32 ± 0.03% to 7.38 ± 0.54%, the minimum was recorded in *E. latifolia* and the maximum in *H. validus*. Our results indicated that the fruits of *H. validus*, *P. nepalensis, B. sapida, E. latifolia, M. esculenta*, and *D. indica* contained higher levels of TSS and acidity. TSS and acidity are the two important factors for determining the quality traits in a fruit, which also influence the taste, sweetness, and also act as an indicator of the maturity of the fruit and its suitability for processing. This was indicated by a strong relationship between TSS and total sugar (0.711^**^), ascorbic acid (0.838^**^), total monomeric anthocyanin (0.732^**^), total carotenoids (0.407*), total flavonoids (0.479^**^), and total flavonol (0.532^**^). Similar observations have been reported by Canan et al. (64). Hence, the fruits rich in TSS and acidity were found suitable for fresh consumption as well as processing and value addition (65), and can be promoted for different value-added products such as ready to serve (RTS), wine, etc. as a cottage industry.

**TABLE 2:** Fruits and seed physical characteristics of promising wild edible fruits grown in the eastern Himalayas, India.

Characters	Fruit length (cm)	Fruit diameter (cm)	Fruit circumference (cm)	Fruit weight (g)	Fruit volume (cm^3^)	Juice content (%)	Pulp (%)	Seed weight (g/seed)	Seed length (mm)	Seed breadth (mm)	Seeds number/Fruits
*Baccaurea sapida*	2.82 ± 0.03^cd^	3.13 ± 0.28^bc^	9.06 ± 0.89^c^	11.86 ± 0.77^cd^	12.39 ± 0.81^ef^	36.3 ± 1.92^b^	56.69 ± 2.5^e^	0.49 ± 0.02^h^	11.35 ± 0.12^e^	9.87 ± 0.07^f^	3.00^c^
*Docynia indica*	4.27 ± 0.14^a^	3.81 ± 0.47^ab^	10.61 ± 1.33^b^	33.17 ± 2.68^a^	33.3 ± 0.12^b^	29.54 ± 2.10^c^	78.54 ± 1.60^b^	0.07 ± 0.01^i^	10.45 ± 0.04^f^	3.16 ± 0.03^h^	4.00^b^
*Elaeagnus latifolia*	3.84 ± 0.39^ab^	2.82 ± 0.35^cd^	8.52 ± 1.06^d^	17.82 ± 0.70^c^	17.92 ± 1.84^d^	28.26 ± 3.86^c^	68.10 ± 1.9^d^	3.37 ± 0.04^a^	32.25 ± 0.37^a^	14.11 ± 0.06^a^	1.00^d^
*Elaeagnus pyriformis*	2.34 ± 0.14^de^	2.04 ± 0.12^de^	6.48 ± 0.38^f^	7.32 ± 0.82^e^	7.32 ± 0.78^g^	29.72 ± 1.53^c^	58.27 ± 0.7^e^	1.06 ± 0.05^f^	20.86 ± 0.06^b^	12.63 ± 0.03^d^	1.00^d^
*Haematocarpus validus*	4.38 ± 0.19^a^	3.08 ± 0.37^bc^	8.58 ± 1.03^d^	23.62 ± 1.76^b^	24.28 ± 0.56^c^	43.72 ± 1.46^a^	76.25 ± 1.86^bc^	2.62 ± 0.02^b^	20.13 ± 0.48^c^	10.58 ± 0.13^e^	1.00^d^
*Myrica esculenta*	3.14 ± 0.15^c^	2.73 ± 0.11^cd^	8.13 ± 0.31d^e^	13.26 ± 1.83^cd^	14.64 ± 1.76^de^	40.36 ± 1.47^ab^	84.67 ± 0.53^a^	2.03 ± 0.06^d^	19.62 ± 0.18^cd^	13.74 ± 0.03^b^	1.00^d^
*Myrica nagi*	1.52 ± 0.05^f^	1.23 ± 0.08^e^	3.64 ± 0.24^g^	8.30 ± 0.56^de^	9.07 ± 0.7^fg^	21.22 ± 0.92^d^	84.12 ± 1.97^a^	1.32 ± 0.06^e^	10.03 ± 0.05^f^	0.82 ± 0.04^i^	1.00^d^
*Prunus nepalensis*	2.14 ± 0.08^e^	2.17 ± 0.02^d^	6.72 ± 0.05^ef^	7.82 ± 0.10^de^	8.23 ± 2.68^g^	26.16 ± 0.98^cd^	71.23 ± 1.47^d^	2.25 ± 0.04^c^	14.36 ± 0.17^d^	13.52 ± 0.07^c^	1.00^d^
*Pyrus pashia*	3.71 ± 0.63^b^	4.39 ± 0.43^a^	12.38 ± 1.13^a^	37.83 ± 4.75^a^	39.89 ± 4.75^a^	29.72 ± 2.95^c^	71.83 ± 1.71^cd^	0.78 ± 0.03^g^	8.72 ± 0.03^g^	4.65 ± 0.04^g^	7.33 ± 24^a^

Values given are mean (*n* = 30) with ± SE followed by different letters on each column indicate significant difference from each other according to Tukey’s test (*p* < 0.05).

**TABLE 3:** Biochemical characteristics of wild edible fruits grown in the eastern Himalayas, India.

Species	Moisture (%)	Total soluble solids (%)	Titratable acidity (%)	Total sugar (%)	Reducing sugar (%)
*Baccaurea sapida*	81.65 ± 2.83^c^	11.97 ± 1.72^c^	1.29 ± 0.06^f^	5.84 ± 0.48^c^	3.72 ± 0.46^d^
*Docynia indica*	76.28 ± 0.5^de^	8.23 ± 0.45^g^	1.32 ± 0.03^f^	5.42 ± 0.11^cd^	3.81 ± 0.18^cd^
*Elaeagnus latifolia*	78.35 ± 2.0^cd^	9.4 ± 0.80^e^	2.68 ± 0.04^b^	3.26 ± 0.05^e^	1.32 ± 0.03^fg^
*E. Pyriformis*	80.49 ± 1.28^bc^	11.33 ± 1.21^cd^	2.23 ± 0.05^d^	3.65 ± 0.05^e^	1.87 ± 0.02^f^
*Haematocarpus validus*	88.39 ± 1.85^a^	18.27 ± 1.49^a^	1.83 ± 0.03^e^	11.27 ± 1.26^a^	7.38 ± 0.54^a^
*Myrica esculenta*	86.75 ± 1.33^ab^	5.83 ± 0.30^hi^	3.32 ± 0.06^a^	4.27 ± 0.08^de^	2.93 ± 0.03^e^
*Myrica nagi*	83.62 ± 1.52^bc^	6.76 ± 0.43^h^	2.45 ± 0.04^c^	6.83 ± 0.05^c^	3.08 ± 0.10^de^
*Prunus nepalensis*	75.26 ± 0.56^e^	16.73 ± 0.93^b^	1.21 ± 0.04^f^	8.74 ± 0.60^b^	4.46 ± 0.20^bc^
*Pyrus pashia*	73.75 ± 1.88^e^	9.38 ± 0.70^ef^	0.31 ± 0.03^g^	6.27 ± 0.49^c^	4.84 ± 0.14^b^

Values given are mean (*n* = 3) with ± SE. One-way analysis of variance (ANOVA) plus *post hoc* Tukey test was done to compare means. Superscript lowercase letters on each column designated statistical significance (*p* < 0.05).

### 3.2. Functional attributes and antioxidant activity

#### 3.2.1. Ascorbic acid content

**FIGURE 3:**
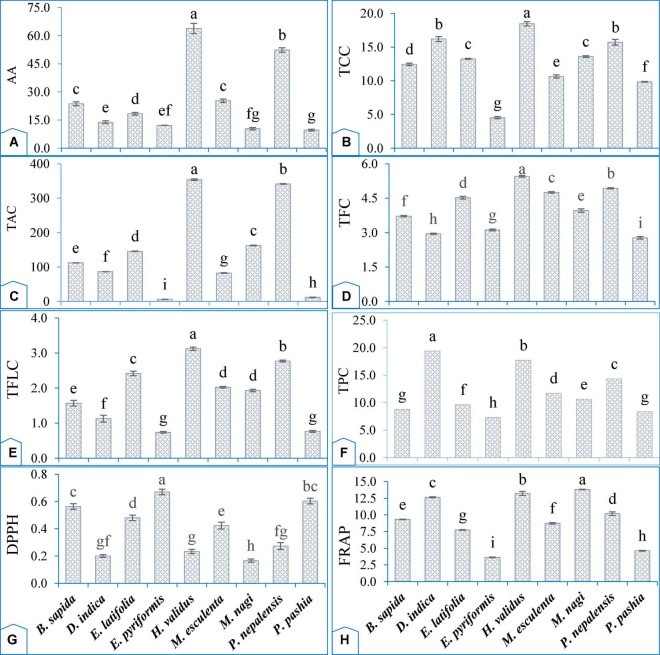
Functional attributes of wild edible fruits grown in the eastern Himalayas, India. **(A)** Ascorbic acid content (AA, mg/100 g fw); **(B)** total carotenoids content (TCC, mg/100 g fw); **(C)** total monomeric anthocyanins content (TAC, mg/100 g fw); **(D)** total flavonoids content (TFC, mg QE/g); **(E)** total flavonol content (TFLC, mg QE/g); **(F)** total phenol content (TPC, mg GAE/g); **(G)** DPPH antioxidants capacity (DDPH, IC_50_ value mg/mL); **(H)** FRAP antioxidants capacity (FRAP, mg AAE/g) content in wild edible fruits. IC_50_ ascorbic acid (0.012 ± 0.002). Mean value of three replications (each replication consisted 10 fruits) with ± S.E followed by different letters on each bar indicate significant difference from each other according to Tukey’s test (*p* < 0.05).

Ascorbic acid is regarded as the most important antioxidant vitamin. However, it cannot be synthesized by humans due to the lack of gulonolactone oxidase enzyme, and a deficiency of dietary ascorbate results in clinical syndrome and scurvy (66). Hence, supplementing the diet with ascorbic acid-rich foods is very vital. In this study, the ascorbic acid content of wild edible fruits had significant variations (Figure 3A). The highest ascorbic acid content was recorded in *H. validus* (63.82 mg/100 g pulp) and the lowest in *P. pashia* (9.62 mg/100 g pulp). These results agree with the reports of Contreras-Calderón et al. (67) on the variability of vitamin C content in several wild edible fruits. Furthermore, the finding demonstrated that these wild edible fruits have a higher vitamin-C content than commercially available major fruits: *Citrus sinensis* (10.13 ± 0.10 mg/100 g), *Ananas comosus* (6.40 ± 0.18 mg/100 g), *Malus domestica* (7.94 ± 0.13 mg/100 g), and *Prunus persica* (5.92 ± 0.12 mg/100 g). However, they had a lesser content than the richest known sources of vitamin C, such as *Psidium guajava* (198.05–221.47 mg/100 g), *Phyllanthus emblica* (375.68 mg/100 g), and *Emblica officinalis* (756.32 mg/100 g) (68–70). Our results showed that the ascorbic acid content of *E. latifolia* was lower than that reported from Sikkim by Dasila and Singh (71). This variation may be attributed to different analytical methods, as reported by Dias et al. (72). The *E. pyriformis* reported in this study had a lower ascorbic acid content than that reported from Manipur (20.10 mg/100 g) by Khomdram et al. (70). The reason for variations may be due to the unique genetic make-up among genotypes and environmental factors (73), and differences in soil physico-chemical attributes such as *p*H, nutrients, and agro-ecology (74). A variation in the pH of the soil is known to determine the availability of nutrients to the roots and their uptake, which could be influenced by soil geology and climatic factors (75). A significant positive correlation of ascorbic acid with total soluble solids (0.838^**^) and total sugar (0.784^**^) was observed (Table 4). A high positive correlation of ascorbic acids with sugars was due to the recurring and elaborate interactions between organic acids and sugars (76), which may be associated with the synthesis of ascorbic acid from glucose (77). Ascorbic acid also showed a significant negative correlation with total antioxidant activity (−0.397*) which was represented by the IC_50_ of DPPH and analyzed by Pearson’s correlation coefficient (r). It is well-established that lower IC_50_ values indicate high antioxidant activity (78). Therefore, an increase in the ascorbic acid content will enhance the antioxidant activity of these fruits, as shown by the lower IC_50_ of DPPH value. Our findings suggested that ascorbic acid may be one of the factors contributing to antioxidant properties, as evidenced by their positive relationship in a variety of other food sources (79). Hence, the daily consumption of these fruit crops will enrich the diet and act as an additional or alternative source of ascorbic acid.

**TABLE 4:** Pearson’s correlation coefficient (r) among biochemical and antioxidant activities of wild edible fruits grown in the eastern Himalayas, India.

Characteristics	TSS	TSG	AA	TAC	TCC	TPC	TFC	TFLC	DPPH	FRAP
TSS	1	0.711**	0.838**	0.732**	0.407*	0.32	0.479**	0.532**	−0.117	0.134
TSG		1	0.784**	0.799**	0.678**	0.551**	0.498**	0.582**	−0.552**	0.563**
AA			1	0.899**	0.622**	0.540**	0.816**	0.821**	−0.397*	0.405*
TAC				1	0.784**	0.567**	0.824**	0.912**	−0.645**	0.635**
TCC					1	0.809**	0.541**	0.709**	−0.818**	0.851**
TPC						1	0.332*	0.431**	−0.794**	0.732**
TFC							1	0.962**	−0.427**	0.447**
TFLC								1	−0.555**	0.569**
DPPH									1	−0.932**
FRAP										1

*Significant at 0.05 level (2-tailed), **significant at 0.01 level (2-tailed). TSS, total soluble solids (%); TSG, total sugar (%); AA, ascorbic acid content (mg/100 g fw); TCC, total carotenoids content (mg/100 g fw); TAC, total anthocyanins content (mg/100 g fw); TFC, total flavonoids content (mg QE/g); TFLC, total flavonol content (mg QE/g); TPC, total phenol content (mg GAE/g); DPPH, DPPH antioxidants capacity (IC_50_ value mg/mL); FRAP, FRAP antioxidants capacity (mg AAE/g).

#### 3.2.2. Total carotenoid content

Carotenoids as antioxidant compounds are known to be present in several fruit crops, and the dietary intake of carotenoid-rich foods has been reported to retard cancer, cardiovascular disease, and several other ailments in humans (80). Results showed that the total carotenoids content of different wild edible fruit species varied significantly (*p* < 0.05; Figure 3B). *H. validus* recorded the highest total carotenoids (18.47 mg/100 g pulp), followed by *D. indica*, and the lowest total carotenoids (4.52 mg/100 g pulp) were noted in *E. pyriformis*. The fruits of *H. validus*, *D. indica*, and *P. nepalensis* contain higher total carotenoids than mangoes [4,926.76–14,942.46 μg/100 g fw, (81)] and cashews [0.4 mg/100 g fw, (82)]. The variations in genetic make-up among the species may be the cause of the variations in total carotenoids. Dias et al. (72) have also reported the great influences of varieties, maturity, cultural management, environment, postharvest care, storage conditions, and analytical methods on the formation of secondary metabolites, including the total carotenoid content in fruit crops. The high carotenoid content of these fruits is an important indicator of their quality and high nutritional value (83). In addition, our study also found a strong negative correlation between total carotenoid content and DPPH (−0.818^**^). The presence of high level of total carotenoids in the fruits of *H. validus, P. nepalensis, D. indica, B. sapida, E. latifolia*, and *M. esculenta* indicates their powerful ability to scavenge oxygen free radicals and active oxygen. The previous study (84) revealed that carotenoid scavenging ability would increase due to an increase in the lipophilicity of carotenoid. Lycopene was effective in reducing Fe (III) to Fe (II), given the fact that lycopene contains 11 conjugated double bonds (84). Although lycopene content was not analyzed in our study, Dasila and Singh (71) found that it was 2.5 times higher in *E. latifolia* (2.06 ± 0.38 mg/100 g) than β-carotene (0.83 ± 0.02 mg/100 g). Our results also indicated that the a* value (redness) of the peel (31.5 ± 3.90*^a^*) and juice (23.4 ± 1.9*^a^*) of *E. latifolia* were the highest among these wild edible fruits (Table 5). It is well established that the red color of certain fruits and vegetables, such as tomato, pink grapefruit, red grapes, watermelon, and red guava, is due to the presence of lycopene (85). Therefore, lycopene may be one of the major pigments responsible for the red color in the fruits of *E. latifolia*.

**TABLE 5:** Color, season of availability and market price of wild edible fruits grown in the eastern Himalayas, India.

Fruits	Peel pigmentation			Juice pigmentation			Season of availability	Yield per tree (kg/tree)	Market price per kg ($)
	L* value	a* value	b* value	L* value	a* value	b* value			
*Baccaurea sapida*	71.8 ± 10.42^a^	−6.500.46^d^	30.9 ± 4.78^a^	35.7 ± 4.75^d^	3.7 ± 0.32^d^	2.5 ± 1.36^f^	June to July	45–138	0.85
*Docynia indica*	54.5 ± 6.80^b^	−6.7 ± 3.77^d^	30.0 ± 2.55^a^	50.3 ± 5.15^bc^	2.4 ± 1.29^d^	15.1 ± 0.71^cd^	November to December	27–125	0.57
*Elaeagnus latifolia*	22.5 ± 2.93^cd^	31.5 ± 3.90^a^	12.9 ± 1.66b^c^	39.8 ± 2.08^cd^	23.4 ± 1.9^a^	17.6 ± 0.71^bc^	March to May	15–117	0.50
*Elaegnus pyriformis*	54.5 ± 2.92^b^	1.8.0 ± 2.03^cd^	17.7 ± 3.27^b^	70.9 ± 8.15^a^	11.1 ± 2.8^c^	20.4 ± 0.40^b^	April to May	7–26	0.21
*Haematocarpus validus*	16.2 ± 4.33^de^	6.3 ± 2.12^c^	−0.1 ± 1.22^d^	14.5 ± 1.56^e^	19.3 ± 3.04^b^	6.5 ± 1.25^e^	June to August	35–83	1.14
*Myrica esculenta*	54.2 ± 6.36^b^	15.7 ± 4.29^b^	29.6 ± 2.05^a^	67.5 ± 2.61^a^	1.7 ± 1.47^e^	17.6 ± 1.29^bc^	June to July	17–116	0.78
*Myrica nagi*	25.6 ± 1.57^cd^	28.5 ± 1.51^a^	9.3 ± 0.67^c^	41.5 ± 3.75^cd^	24.6 ± 2.95^a^	13.4 ± 1.21^d^	June to July	12–53	0.99
*Prunus nepalensis*	15.8 ± 1.30^e^	2.6 ± 0.44^cd^	−0.7 ± 0.71^d^	8.2 ± 1.21^e^	14.1 ± 2.15^bc^	4.3 ± 1.90^ef^	August to October	20–125	1.70
*Pyrus pashia*	33.2 ± 1.33^c^	20.1 ± 1.91^b^	18.1 ± 0.95^b^	61.1 ± 2.21^bc^	0.7 ± 0.29^d^	31.2 ± 0.55^a^	August to October	40–132	0.64

Mean value of three replications (each replication consisted 10 fruits) with ± S.E followed by different letters on each bar indicate significant difference from each other according to Tukey’s test (*p* < 0.05). Based price of 2019, 1 USD = 70.39 INR.

#### 3.2.3. Total monomeric anthocyanin content

Anthocyanins are water-soluble and vacuolar pigments found in most species in the plant kingdom. Its accumulation mostly occurs on flowers and fruits, which impart an attractiveness to the fruit, hence; it is considered a color indicator and a natural colorant (86). It also plays a role in preventing, ameliorating, and scrubbing oxidative stress, thus retarding several diseases and physiological malfunctions (87). A significant variation (*p* < 0.05) in total monomeric anthocyanin content was observed among wild edible fruits (Figure 3C). The highest total monomeric anthocyanin content was recorded in *H. Validus* (354.04 mg/100 g), followed by *P. nepalensis* (341.70 mg/100 g) and the lowest was found in *E. pyriformis* (6.02 mg/100 g). These wild fruits contain more total monomeric anthocyanin than commercial fruit cultivars such as sweet cherry *cv*. Black Gold [44.19 ± 1.38 mg/100 g fw, (88)], red currants (12.14 ± 0.87 mg/100 g fw), black currant (287.78 ± 0.08 mg/100 g fw) (89), purple tomato [20.73 ± 2.86 mg/100 g fw, (90)], and guava [0.40–0.69 mg/100 g, (82)]. The varied total anthocyanin levels between species indicate genetic variations in the synthesis of these bioactive substances. Horbowicz et al. (86) have also reported the considerable variation in anthocyanin content of the fruits among different species or cultivars within the same species. This difference in anthocyanin content among these fruits might be due to the effects of genetics, agro-ecological conditions such as pH, light, temperature, and horticultural practices (91). In our study, it was also observed that the fruits with higher anthocyanin content, such as *H. Validus* (−0.7 ± 0.71) and *P. nepalensis* (−0.1 ± 1.22) had the lowest b* value (Table 5). Similarly, the lowest L* values were recorded in the darkest colored fruits (*H. Validus*, 16.2 ± 4.33 and *P. nepalensis*, 15.8 ± 1.30). In the previous study by Muzolf-Panek and Waskiewicz (92), it was revealed that the effect of variety was predominant in fruit peel color and that the darkest table grapes had the lowest L* values, indicating blue-black and violet-black peel in varieties of table grapes. According to Ponder et al. (93), anthocyanins are responsible for the specific dark blue color of fruit berries, and the darker the fruit, the more anthocyanins it contains. Therefore, the dark purple and blue fruit color of *H. validus* and *P. nepalensis* might be due to their high anthocyanin content. Furthermore, a significant inverse relationship (−0.645^**^) between total monomeric anthocyanin content and DPPH demonstrated their high antioxidant properties. Similarly, Katiresh et al. (94) found that anthocyanins in *Sesbania sesban* had high antioxidant activity and were effective at scavenging free radical DPPH. Structurally, monomeric anthocyanin possesses loose structures that are easier to undergo oxidation and thus will exhibit better antioxidant activity compared to non-monomeric anthocyanin (95). This is also in agreement with Castaneda-Ovando et al. (96), who claimed that the molecule that donates a free electron (ionization potential) or hydrogen atoms (bond dissociation energy) to the reactive free radicals is often the best antioxidant, and increasing the stability of the anthocyanin will reduce its antioxidant stability. As a result, consuming fruits with high concentrations of these compounds may provide protection to the body against various illnesses (97).

#### 3.2.4. Total flavonoids and flavonols

Flavonoids and flavonols are naturally occurring phenolic compounds found in fruits, vegetables, and/or medicinal plants. They have significant biological effects and exhibit promising antioxidant activity due to their ability to effectively scavenge reactive oxygen species. Dietary flavonoids are recognized for their antioxidant potential, antiproliferative effects, and protective effects on lipids and vital cells against oxidative damage. These properties also play a significant role in the prevention of cardiovascular disease, inflammation, and antiproliferative or anticancer activities (98). A significant variation was also recorded in total flavonoids and flavonol content among different wild fruit species (*p* < 0.05, Figures 3D, E). Total flavonoid content values ranged from 2.77 ± 0.06 mg QE/g (*P. pashia*) to 5.46 ± 0.04 mg QE/g (*H. validus*). Similarly, total flavonol content also varied significantly among the studied fruits, being the maximum in *H. validus* (3.12 ± 0.05 mg QE/g) and the minimum in *E. pyriformis* (0.74 ± 0.03 mg QE/g). These fruits contained a higher level of flavonoids than most of the plants reported by Fouad et al. (99) and also higher concentrations of flavonols than *Prunus mahaleb* [1.24 ± 0.06 g/kg, (100)]. This variation in total flavonoids and flavonol content among different fruit species could be due to various intrinsic and extrinsic factors, such as genetic and environmental factors. Our results revealed a strong negative correlation of total flavonoids content (−0.794^**^) and total flavonol content (−0.427^**^) with DPPH content, which indicates that flavonoids and flavonols play an important role in the antioxidant activity of these fruits. Our study is in line with that of Chandra et al. (101), who reported that 32% of the antioxidant activity in crops was contributed by flavonoids, which constitute a major group of antioxidant compounds and act as primary antioxidants (102). The redox properties of total flavonoids were due to the unique positions of OH ortho (C-3′ and C-4′) and oxo functional groups (C-4) in flavonoids (103). Therefore, fruit trees such as *H. validus*, *M. esculenta, B. Sapida, M. nagi, E. latifolia, D. indica*, and *P. nepalensis* are rich in flavonoids and flavonol content, suggesting their consumption can help people meet their nutritional needs and protect them from developing a variety of degenerative diseases.

#### 3.2.5. Total phenolic content

Plant-derived phenolic compounds are a diverse group of secondary metabolites that interact with reactive oxygen species to prevent oxidative damage, thereby aiding plant defense mechanisms and protecting humans from a variety of degenerative diseases (104). Our results indicated the presence of significant variations (*p* < 0.05) in total phenolic content among fruits in the following descending order: *D. indica* (19.37 ± 0.07 mg GAE/g) followed by *H. validus, P. nepalensis, M. esculenta, M. nagi*, *E. latifolia, B. sapida, P. pashia*, and *E. Pyriformis* (7.32 ± 0.11 mg GAE/g) (Figure 3F). Interestingly, fruits like *D. indica, H. validus, P. nepalensis, M. esculenta, M. nagi*, and *E. latifolia* had higher total phenolic content than the commercial crops of the region, such as pineapple (47.9 mg GAE/100 g), banana (7.2 ± 0.5–18.9 ± 1.4 mg GAE/g dw), and papaya (57.6 mg GAE/100 g) (105, 106). These fruits are comparable with the known richest sources of total phenolic content, such as Aonla (944.85–4,969.50 mg/100 g pulp), which are grown locally (107). According to Robards et al. (108), phenolic compounds exhibit heterogeneity in distribution and concentration across and within plant species. Furthermore, the higher phenol accumulation in the fruits under our study might depend on several factors, *viz*., agroclimatic conditions, organ, plant developmental stage, and their interaction with the genotype (10). The presence of different concentrations of sugars, carotenoids, or ascorbic acid, as well as extraction methods, may influence the amount of phenolics (109). Czyczyło-Mysza et al. (110) also suggested the importance of both additive and epistatic gene effects on total phenolic content in species, which affect other adaptation traits of the species. Total phenolic content had a positive correlation with total sugar (0.551^**^), ascorbic acid, DPPH (−0.794^**^), total monomeric anthocyanin, total carotenoids, total flavonoids, and total flavonol. According to Fitriansyah et al. (111), if the r value is −0.61 ≤ *r* ≤ −0.9723, it showed a high negative correlation, which indicates that TPC had a strong negative correlation with DPPH. It is well established that phenolic compounds are important plant constituents with redox properties responsible for antioxidant activity. The higher the TPC, the greater is the total antioxidant activity of these fruits as demonstrated by low IC_50_ of DPPH. Our study exhibits that TPC was one of the major contributory compounds for antioxidant activity, which was also confirmed by Nariya et al. (112) for their scavenging ability due to their unique hydroxyl groups. This indicates that these wild food resources are highly nutritious and rich sources of bioactive compounds, and their consumption will further help improve nutrition.

#### 3.2.6. DPPH free radical scavenging activity

The free radical chain reaction is widely accepted as the most important mechanism of lipid peroxidation. Radical scavengers terminate the peroxidation chain reaction by directly counteracting and quenching peroxide radicals. The capacity of polyphenols to transport labile H atoms to radicals is a probable mechanism of antioxidant protection, which can be assessed universally and rapidly using DPPH. Furthermore, DPPH is the most common and cost-effective way to determine the free radical scavenging capacity of natural products, which are major factors in biological damage caused by oxidative stress (113). Our results revealed a significant variation (*p < 0.05*) in DPPH free radical scavenging activity among the studied fruits (Figure 3G), and it ranges from (0.17 ± 0.01 IC_50_ mg/mL) in *M. nagi* to 0.67 ± 0.02 IC_50_ mg/mL in *E. pyriformis*. The present results showed lesser values than those recorded in commercial fruits such as grapes (0.79 ± 0.34 IC_50_ mg/mL), pineapple (0.83 ± 0.24 IC_50_ mg/mL), and guava (1.71 ± 0.61 IC_50_ mg/mL) (79). The antioxidant capacity of fruits and vegetables was influenced by factors such as genetic makeup, maturity, and other environmental factors such as sunlight exposure, soil, and the gene-environment interaction (10). According to Matuszewska et al. (78), the lower IC_50_ values of DPPH indicate a high level of antioxidant activity, which means that these fruits, *viz*., *M. nagi, D. indica, H. validus*, and *P. nepalensis* with a low IC_50_ value can scavenge the DPPH radicals to form a stable reduced DPPH molecule. The high accumulation of total sugar, ascorbic acid, total monomeric anthocyanin, total carotenoids, total phenolics, total flavonoids, and total flavonol content increases the antioxidant activity, as demonstrated by the lower IC_50_ of DPPH value in our result, which agreed with the finding of Sundaramoorthy and Packiam (114). Therefore, the high antioxidant activity of these fruits might be due to a strong negative correlation of different compounds with IC_50_ DPPH. Previous studies have also found that the antioxidant activity in plant tissue was mainly due to the unusual redox properties of not just one particular compound but also of different bioactive compounds including TPC, tannin, anthocyanin, TFC, phenols, alkaloids, and pro-anthocyanins (115, 116). The antioxidant effect is due to the ability of compounds in the plant extract to transfer electrons or hydrogen atoms to neutralize radicals of DPPH and form neutral DPPH molecules (117). Hence, it is clear from our results that these fruit crops had a greater potential for radical scavenging compounds with proton-donating abilities.

#### 3.2.7. FRAP reducing power

FRAP antioxidants capacity is a simple and inexpensive assay that offers a putative index of the potential antioxidant activity of plant materials. Principally, the FRAP assay treats the antioxidants in the sample as reductants in a redox-linked colourimetric reaction. The reducing power assay, i.e., the transformation of Fe^3+^ to Fe^2+^ in the presence of either the extract or the standard (ascorbic acid), is a measure of reducing capability (79). A significant variation (*p < 0.05*) of FRAP reducing power was observed among the underutilized fruits studied, and it ranged from 3.63 ± 0.05 mg AAE/g in *E. pyriformis* to 13.82 ± 0.04 mg AAE/g in *M. nagi* (Figure 3H). These results indicated higher FRAP values in these fruits than in the other wild fruits reported (0.0518 ± 0.49 to 0.111 ± 0.00 mg AAE/g) by Mahadkar et al. (118) from central India. The differences in antioxidant content between species may be due to genetics and environmental factors, as well as their interactions. Our result showed a strong positive correlation of FRAP value with total sugar (0.563^**^), ascorbic acid (0.405*), total monomeric anthocyanin (0.635^**^), total carotenoids (0.851^**^), total phenolics (0.732^**^), total flavonoids (0.447^**^), total flavonol (0.569^**^), and inversely related to DPPH (IC_50_, −0.932^**^) (Table 4). The finding that the DPPH and FRAP assays of fruit extracts were highly correlated agrees with the work of Szydłowska-Czerniak et al. (119) and is consistent with the view that the two assays share a similar mechanistic basis, *viz*., transfer of electrons from the antioxidant to reduce an oxidant, as proposed by Huang et al. (120). The total carotenoids and total phenolic content were the major compounds contributing to the antioxidant activity in our study. Previous report in amla fruits (*Emblica officinalis* Gaertn) showed that carotenoids had reduction potential lower than 0.44 V, allowing them to reduce Fe (III) to Fe (II) while also being oxidized and acting as antioxidants (84). Similarly, the phenolic compounds largely contribute to the antioxidant activities of these species and therefore could play an important role in the beneficial effects of these fruits. Several studies have found that phenolic compounds are major antioxidant constituents in selected plants and that there are direct relationships between their antioxidant activity and total phenolic content (103). The antioxidant properties of phenolic compounds are directly linked to their unique structure, which allows them to act as reducing agents, hydrogen donors, and singlet oxygen quenchers (121). This demonstrated that most of these underutilized fruits have strong reducing capabilities as compared to other fruit crops, which might be due to the presence of high total carotenoids, total phenolic content, and other functional compounds that are responsible for their antioxidant activity (122). In general, among the fruits, *M. nagi* showed relatively stronger FRAP activity than other fruits.

### 3.3. Color, season of availability and market price of fruits

**FIGURE 4:**
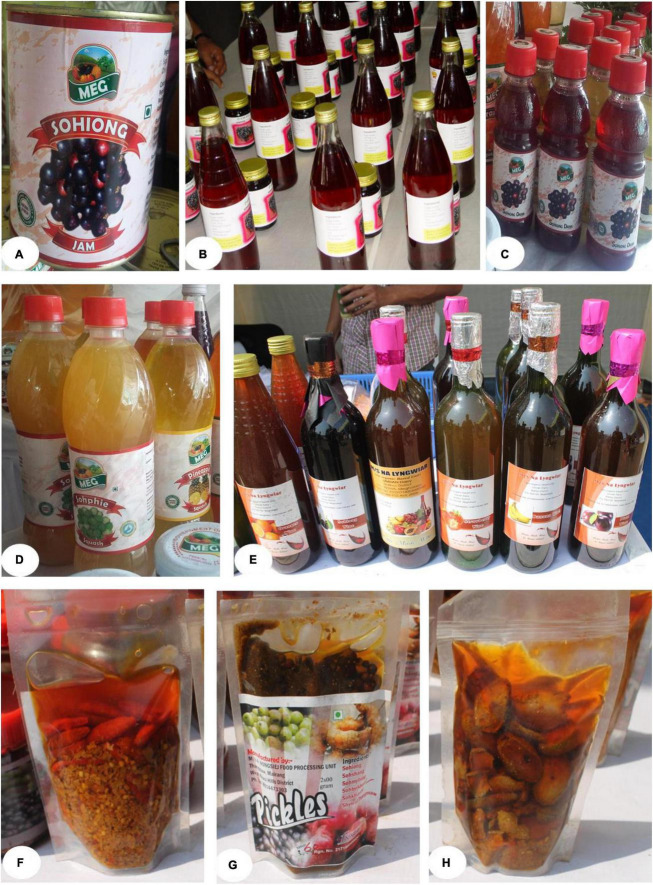
Different products developed by farmers from wild edible fruits grown in the eastern Himalayas, India. **(A)** Jam of Sohiong (*P. nepalensis* Ser.); **(B)** Squash of *P. nepalensis*; **(C)** Juice of *P. nepalensis*; **(D)** Juice of Sohphie (*M. esculenta*); **(E)** Wine of *P. nepalensis*; **(F)** pickle of Sohphoh (*D. indica*); **(G)** Mixed pickles of *P. nepalensis* and *E. latifolia*; **(H)** pickle of Sohshang (*E. latifolia*).

These fruits have appealing pigments, both in the peel and juice, as evidenced by a significant variation in value of L*, a* and b* in both peel and juice color (*p* < 0.05, Table 5). *B. sapida* had the highest peel L* value (71.80 ± 10.42a) and b* value (30.90 ± 4.78a), while *E. latifolia* had the highest peel a* value (31.50 ± 3.90a). Similarly, the yellowness and redness of these fruits were found to be higher than many of the Indian commercial mango varieties (123). The color variation (L*, a, b) among the wild edible fruit trees might be due to a genetic effect. It is well established that the L* value is a suitable indicator of darkening that arises either from increasing pigment concentrations or from oxidative browning reactions (124). Furthermore, the higher a* and b* values added a decorative effect toward the consumer’s preference. These fruits can be a good potential source for the extraction of natural edible color that is required in the food industry. As per Deka et al. (125), the products prepared from *P. nepalensis*, such as squash and jam, develop an attractive color and remain stable for 1 year (Figure 4). They have also prepared ready-to-serve (RTS) products and cherry wine from *P. nepalensis* fruits, which impart a unique natural purple color (13). The suitability of products for processing and extracting natural color helps stabilize the market price. The results also showed that the season of availability of fruits varies from plant to plant. The fruits of these wild edible fruit plants were found to be available throughout the year, with the exception of January and February. The different harvesting periods of these wild edible fruits ensure the year-round availability of these fruits, and particularly during the lean season when other fruits are not available, they provide supplementary food and nutritional security in the region. The yield of wild edible fruit trees varies between 7–26 kg per tree in *E. pyriformis* and 45–138 kg per tree in *B. sapida*. The variation in season of fruit availability and yield among wild edible fruit trees might be due to the contribution of genetic makeup and the growing environment (126). Similarly, the market price of fruits ranged from $ 0.21 per kg in *E. pyriformis* to $ 1.7 per kg in *P. nepalensis*. The variation in market price among wild edible fruits might be due to the contribution of fruit quality factors such as taste, TSS-acidity blend, peel appearance, etc., which determine the appealability and preferences amongst consumers. Tarancon et al. (127) also reported that the consumer’s perception of fruit quality is exclusively based on appearance. The market price of the fruits of *P. nepalensis* was about $34.10–$141.5 per tree, which highlights the high potential for income generation from these wild fruit trees. Therefore, expansion of the commercial area under these crops and their utilization may offer an additional source of income, employment generation, and livelihood improvement.

## 4. Conclusion

About 12% of the world’s population lives in mountainous regions. Wild edible fruits have been consumed by the mountainous populace since time immemorial. Many of these genetic resources, however, have become rare and endangered as a result of overexploitation in their natural habitat and a lack of consumer understanding of their antioxidant and biochemical values. Our results would aid in a proper understanding of the potential uses and antioxidant activities of wild edible fruit trees. Therefore, it is concluded that:

Wild fruits such as *H. validus, P. nepalensis, B. sapida, E. latifolia, M. esculenta, and D. indica* are high in total soluble solids, total sugar, and acidity. These fruits have the potential to be used as supplementary bases in the fruit processing industry.

The high antioxidant activities such as ascorbic acid, total phenolic content, total flavonoid, total flavonol, DPPH free scavenging capacity, and FRAP reducing power in *H. validus, P. nepalensis, M. esculenta*, and *M. nagi* suggest their potential as sources of bioactive compounds.

These fruits can be used to extract attractive natural colors and to make high-value processed products such as jams, squash, pickles, and wine.

A proper understanding of the biochemical and antioxidant properties of these fruits will help in their sustainable utilization and conservation.

## Data availability statement

The original contributions presented in this study are included in the article/supplementary material, further inquiries can be directed to the corresponding author.

## Author contributions

HR: laboratory studies, design of the figures, and writing the manuscript. VV: data analysis and interpretation of finding. HT, V, RS, and KB: editing of the manuscript. SA, MD, LC, and BM: correction of manuscript. JM, ARS: data collections, laboratory studies, and design of the figures. SH and VM: discussion of the results, critical feedback, and providing help in shaping the content, and evaluation of the manuscript. All authors contributed to the article and approved the submitted version.
